# 3-Bromo-1-(3-chloro­pyridin-2-yl)-*N*-(4-eth­oxy­phen­yl)-1*H*-pyrazole-5-carbox­amide

**DOI:** 10.1107/S1600536810040158

**Published:** 2010-10-30

**Authors:** Hai Yue, Wei-Li Dong, Run-Ling Wang, Xian-Chao Cheng

**Affiliations:** a School of Pharmacy, Tianjin Medical University, Tianjin 300070, People’s Republic of China

## Abstract

In the title compound, C_17_H_14_BrClN_4_O_2_, the pyrazole ring is almost coplanar with the benzene ring [dihedral angle = 0.5 (2)°], whereas the pyrazole ring is close to perpendicular to the 3-chloro­pyridine ring [dihedral angle = 73.7 (2)°]. An intra­molecular C—H⋯O hydrogen bond occurs. The dominant inter­action in the crystal packing is an N—H⋯N hydrogen bond, which generates a chain along the *c* axis. Weak inter­molecular C—H⋯O and C—H⋯N contacts are also observed

## Related literature

For details of the synthesis, see: Dong *et al.* (2009 ▶). For the biological activity of related compounds, see: Gewehr *et al.* (2007 ▶); Dong *et al.* (2008*a* ▶,*b* ▶); Liu *et al.* (2007 ▶); Liu *et al.* (2009*a* ▶,*b* ▶,*c* ▶).
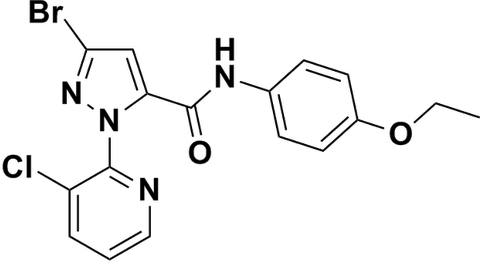


## Experimental

### 

#### Crystal data

C_17_H_14_BrClN_4_O_2_
*M* *_r_* = 421.68
Monoclinic, 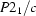

*a* = 16.821 (3) Å
*b* = 10.195 (2) Å
*c* = 10.064 (2) Å
β = 101.09 (3)°
*V* = 1693.7 (6) Å^3^
*Z* = 4
Mo *K*α radiation
μ = 2.60 mm^−1^
*T* = 113 K
0.16 × 0.12 × 0.08 mm

#### Data collection

Rigaku Saturn diffractometer
Absorption correction: multi-scan (*CrystalClear*, Rigaku/MSC, 2002 ▶) *T*_min_ = 0.681, *T*_max_ = 0.819
11217 measured reflections
2967 independent reflections
2459 reflections with *I* > 2σ(*I*)
*R*_int_ = 0.049

#### Refinement

*R*[*F* ^2^ > 2σ(*F* ^2^)] = 0.029
*wR*(*F* ^2^) = 0.064
*S* = 0.99
2967 reflections
231 parameters
1 restraint
H atoms treated by a mixture of independent and constrained refinement
Δρ_max_ = 0.44 e Å^−3^
Δρ_min_ = −0.37 e Å^−3^

### 

Data collection: *CrystalClear* (Rigaku/MSC, 2002 ▶); cell refinement: *CrystalClear*; data reduction: *CrystalClear*; program(s) used to solve structure: *SHELXS97* (Sheldrick, 2008 ▶); program(s) used to refine structure: *SHELXL97* (Sheldrick, 2008 ▶); molecular graphics: *SHELXTL* (Sheldrick, 2008 ▶); software used to prepare material for publication: *SHELXTL*.

## Supplementary Material

Crystal structure: contains datablocks global, I. DOI: 10.1107/S1600536810040158/kp2276sup1.cif

Structure factors: contains datablocks I. DOI: 10.1107/S1600536810040158/kp2276Isup2.hkl

Additional supplementary materials:  crystallographic information; 3D view; checkCIF report

## Figures and Tables

**Table 1:** Hydrogen-bond geometry (Å, °)

*D*—H⋯*A*	*D*—H	H⋯*A*	*D*⋯*A*	*D*—H⋯*A*
N1—H1⋯N4^i^	0.88 (1)	2.21 (1)	3.059 (3)	165 (2)
C2—H2⋯O2	0.93	2.26	2.850 (3)	121
C6—H6⋯N4^i^	0.93	2.60	3.358 (3)	140
C8—H8*A*⋯O2^ii^	0.96	2.45	3.397 (3)	168
C11—H11⋯N4^i^	0.93	2.55	3.390 (3)	151
C16—H16⋯O2^iii^	0.93	2.33	3.244 (3)	167

Symmetry codes: (i) 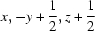; (ii) 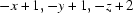; (iii) 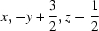.

**Fig. 1.**
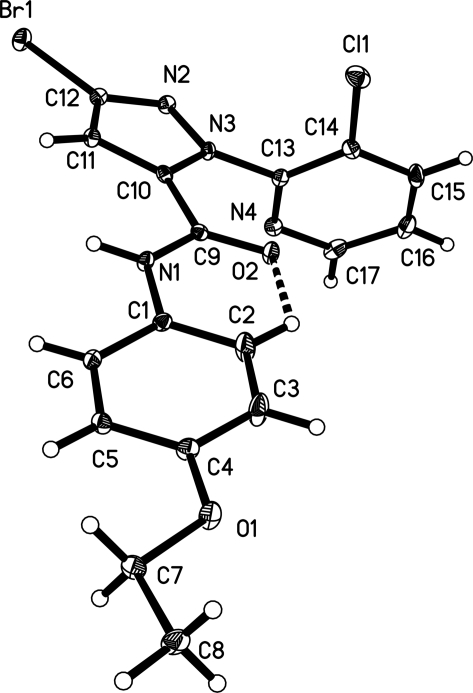
The molecular structure of (I) with intramolecular C—H···O interaction, showing displacement ellipsoids drawn at the 30% probability level.

**Fig. 2.**
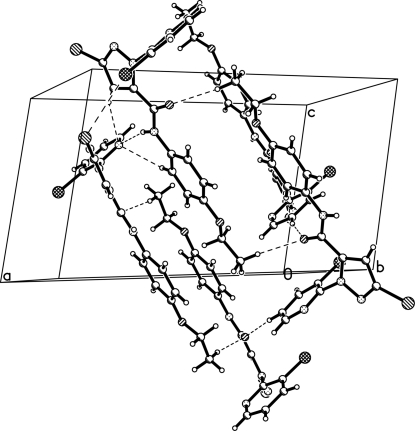
The crystal packing of I with hydrogen bonds shown (in dashed lines).

**Fig. 3.**
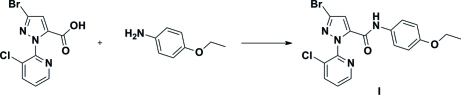
The formation of the title compound.

## supplementary crystallographic information

### Comment

Due to the capability of insects to rapidly develop resistance, the discovery of agents that act on new biochemical targets is an important tool for effective pest management. Calcium channels, in particular, the ryanodine receptors (RyR) represent an attractive biological target for insect control and thus offer excellent promise in integrated pest management strategies.

Many pesticide contain amide structures (Liu *et al.* 2007; Dong *et al.* 2008*a*,*b*; Liu *et al.* 2009*a*,*b*,*c*). Recently, diamides have attracted considerable attention in the field of agrochemistry, owing to their prominent insecticidal activity (Gewehr *et al.* 2007), unique modes of action and good environmental profiles.

Thus, a series of novel amides containing *N*-pyridylpyrazole were synthesized and their insecticidal activities were tested. Here we present the crystal structure of the title compound,(I), which has been determined during a search for relationships between the structure and insecticidal activity of the above derivatives.

The molecular structure of title compound (Fig.1) reveals that the pyrazole ring is coplanar with the benzene ring [dihedral angle 179.5 (2)°] whereas the pyrazole ring is almost perpendicular to 3-chlorpyridine ring [dihedral angle 106.3 (2)°]. In the crystal packing dominating N—H···N interaction and weak C—H···O and C—H···N contacts were observed (Table 1, Fig. 2).

### Experimental

Compound (I) was prepared according to the reported procedure of Dong *et al.*(2009). Colourless single crystals suitable for X-ray diffraction analysis were obtained by recrystallization from ethanol.

### Refinement

H atom of N—H was located in a difference map and refined freely. All C—H atoms were generated by riding model with C—H distance fixed at 0.93(phenyl group), 0.97(methylene group).

### Crystal data

**Table:**

C_17_H_14_BrClN_4_O_2_	*F*(000) = 848
*M**_r_* = 421.68	*D*_x_ = 1.654 Mg m^−^^3^
Monoclinic, *P*2_1_/*c*	Mo *K*α radiation, λ = 0.71073 Å
Hall symbol: -P 2ybc	Cell parameters from 4811 reflections
*a* = 16.821 (3) Å	θ = 2.0–27.1°
*b* = 10.195 (2) Å	µ = 2.60 mm^−^^1^
*c* = 10.064 (2) Å	*T* = 113 K
β = 101.09 (3)°	Ractangle, colourless
*V* = 1693.7 (6) Å^3^	0.16 × 0.12 × 0.08 mm
*Z* = 4	

### Data collection

**Table:**

Rigaku Saturn diffractometer	2967 independent reflections
Radiation source: rotating anode	2459 reflections with *I* > 2σ(*I*)
confocal	*R*_int_ = 0.049
ω scans	θ_max_ = 25.0°, θ_min_ = 2.4°
Absorption correction: multi-scan (*CrystalClear*, Rigaku/MSC, 2002)	*h* = −14→20
*T*_min_ = 0.681, *T*_max_ = 0.819	*k* = −11→12
11217 measured reflections	*l* = −11→11

### Refinement

**Table:**

Refinement on *F*^2^	Primary atom site location: structure-invariant direct methods
Least-squares matrix: full	Secondary atom site location: difference Fourier map
*R*[*F*^2^ > 2σ(*F*^2^)] = 0.029	Hydrogen site location: inferred from neighbouring sites
*wR*(*F*^2^) = 0.064	H atoms treated by a mixture of independent and constrained refinement
*S* = 0.99	*w* = 1/[σ^2^(*F*_o_^2^) + (0.0325*P*)^2^] where *P* = (*F*_o_^2^ + 2*F*_c_^2^)/3
2967 reflections	(Δ/σ)_max_ = 0.002
231 parameters	Δρ_max_ = 0.44 e Å^−^^3^
1 restraint	Δρ_min_ = −0.37 e Å^−^^3^

### Special details

**Table:**

Geometry. All e.s.d.'s (except the e.s.d. in the dihedral angle between two l.s. planes) are estimated using the full covariance matrix. The cell e.s.d.'s are taken into account individually in the estimation of e.s.d.'s in distances, angles and torsion angles; correlations between e.s.d.'s in cell parameters are only used when they are defined by crystal symmetry. An approximate (isotropic) treatment of cell e.s.d.'s is used for estimating e.s.d.'s involving l.s. planes.
Refinement. Refinement of *F*^2^ against ALL reflections. The weighted *R*-factor *wR* and goodness of fit *S* are based on *F*^2^, conventional *R*-factors *R* are based on *F*, with *F* set to zero for negative *F*^2^. The threshold expression of *F*^2^ > σ(*F*^2^) is used only for calculating *R*-factors(gt) *etc*. and is not relevant to the choice of reflections for refinement. *R*-factors based on *F*^2^ are statistically about twice as large as those based on *F*, and *R*- factors based on ALL data will be even larger.

### Fractional atomic coordinates and isotropic or equivalent isotropic displacement parameters (Å^2^)

**Table:**

	*x*	*y*	*z*	*U*_iso_*/*U*_eq_	
Br1	0.023579 (14)	0.06344 (2)	0.32526 (2)	0.02098 (9)	
Cl1	0.07553 (4)	0.63129 (7)	0.54321 (6)	0.02706 (17)	
O1	0.49241 (10)	0.39661 (16)	1.23908 (18)	0.0251 (4)	
O2	0.25436 (9)	0.48804 (16)	0.66299 (16)	0.0182 (4)	
N1	0.26429 (11)	0.29027 (19)	0.77115 (19)	0.0143 (5)	
N2	0.09269 (10)	0.31241 (18)	0.35049 (19)	0.0148 (5)	
N3	0.14508 (10)	0.38461 (18)	0.44293 (18)	0.0125 (4)	
N4	0.21996 (11)	0.49665 (19)	0.3078 (2)	0.0171 (5)	
C1	0.32339 (13)	0.3197 (2)	0.8882 (2)	0.0147 (5)	
C2	0.34708 (16)	0.4465 (2)	0.9280 (3)	0.0292 (7)	
H2	0.3250	0.5177	0.8759	0.035*	
C3	0.40344 (16)	0.4662 (3)	1.0450 (3)	0.0318 (8)	
H3	0.4189	0.5514	1.0712	0.038*	
C4	0.43755 (13)	0.3629 (2)	1.1245 (2)	0.0181 (6)	
C5	0.41491 (13)	0.2361 (2)	1.0851 (2)	0.0162 (6)	
H5	0.4372	0.1650	1.1372	0.019*	
C6	0.35841 (13)	0.2165 (2)	0.9668 (2)	0.0165 (6)	
H6	0.3437	0.1313	0.9398	0.020*	
C7	0.53387 (14)	0.2914 (2)	1.3173 (2)	0.0204 (6)	
H7A	0.5647	0.2407	1.2631	0.024*	
H7B	0.4954	0.2336	1.3481	0.024*	
C8	0.58986 (14)	0.3521 (3)	1.4367 (3)	0.0257 (7)	
H8A	0.6282	0.4079	1.4048	0.039*	
H8B	0.6182	0.2841	1.4925	0.039*	
H8C	0.5588	0.4031	1.4886	0.039*	
C9	0.23427 (13)	0.3734 (2)	0.6695 (2)	0.0128 (5)	
C10	0.17324 (13)	0.3141 (2)	0.5591 (2)	0.0133 (5)	
C11	0.13590 (13)	0.1945 (2)	0.5421 (2)	0.0141 (5)	
H11	0.1412	0.1253	0.6033	0.017*	
C12	0.08813 (13)	0.1993 (2)	0.4129 (2)	0.0143 (5)	
C13	0.17110 (13)	0.5071 (2)	0.3970 (2)	0.0144 (5)	
C14	0.14284 (13)	0.6260 (2)	0.4354 (2)	0.0173 (6)	
C15	0.16840 (15)	0.7398 (2)	0.3811 (3)	0.0247 (7)	
H15	0.1510	0.8214	0.4056	0.030*	
C16	0.21971 (15)	0.7303 (2)	0.2907 (3)	0.0257 (7)	
H16	0.2381	0.8052	0.2532	0.031*	
C17	0.24346 (14)	0.6071 (3)	0.2566 (2)	0.0226 (6)	
H17	0.2777	0.6011	0.1945	0.027*	
H1	0.2473 (12)	0.2088 (11)	0.765 (2)	0.013 (6)*	

### Atomic displacement parameters (Å^2^)

**Table:**

	*U*^11^	*U*^22^	*U*^33^	*U*^12^	*U*^13^	*U*^23^
Br1	0.02471 (15)	0.01863 (15)	0.01646 (15)	−0.00563 (10)	−0.00392 (10)	−0.00200 (11)
Cl1	0.0241 (3)	0.0302 (4)	0.0266 (4)	0.0044 (3)	0.0042 (3)	−0.0100 (3)
O1	0.0317 (10)	0.0178 (10)	0.0183 (10)	−0.0035 (7)	−0.0142 (8)	0.0027 (8)
O2	0.0232 (9)	0.0126 (9)	0.0163 (9)	−0.0023 (7)	−0.0023 (7)	0.0005 (7)
N1	0.0168 (10)	0.0128 (11)	0.0113 (11)	−0.0031 (8)	−0.0022 (8)	−0.0001 (9)
N2	0.0165 (10)	0.0159 (11)	0.0107 (11)	−0.0010 (8)	−0.0011 (8)	−0.0029 (9)
N3	0.0162 (10)	0.0103 (10)	0.0101 (10)	−0.0011 (8)	0.0000 (8)	−0.0013 (8)
N4	0.0183 (11)	0.0175 (12)	0.0133 (11)	0.0002 (9)	−0.0025 (8)	−0.0013 (9)
C1	0.0139 (12)	0.0157 (13)	0.0136 (13)	−0.0017 (10)	0.0000 (10)	0.0007 (10)
C2	0.0378 (16)	0.0161 (15)	0.0250 (16)	0.0011 (11)	−0.0154 (12)	0.0036 (12)
C3	0.0452 (18)	0.0155 (14)	0.0260 (16)	−0.0053 (12)	−0.0145 (13)	−0.0015 (12)
C4	0.0191 (13)	0.0200 (14)	0.0127 (13)	−0.0019 (10)	−0.0034 (10)	−0.0002 (11)
C5	0.0185 (13)	0.0163 (13)	0.0124 (14)	0.0016 (10)	0.0000 (10)	0.0036 (10)
C6	0.0160 (12)	0.0133 (13)	0.0191 (15)	−0.0016 (9)	0.0006 (10)	−0.0003 (10)
C7	0.0213 (13)	0.0219 (14)	0.0153 (14)	0.0016 (10)	−0.0031 (11)	0.0036 (11)
C8	0.0240 (14)	0.0275 (16)	0.0209 (15)	−0.0052 (11)	−0.0078 (11)	0.0032 (12)
C9	0.0127 (12)	0.0136 (13)	0.0121 (13)	0.0016 (10)	0.0027 (9)	−0.0021 (10)
C10	0.0148 (12)	0.0129 (13)	0.0113 (13)	0.0029 (9)	0.0004 (10)	0.0025 (10)
C11	0.0173 (13)	0.0142 (13)	0.0109 (13)	0.0000 (10)	0.0025 (10)	0.0028 (10)
C12	0.0151 (12)	0.0155 (14)	0.0124 (13)	−0.0011 (10)	0.0029 (10)	−0.0013 (10)
C13	0.0153 (12)	0.0144 (13)	0.0104 (13)	−0.0012 (10)	−0.0050 (10)	0.0017 (10)
C14	0.0180 (13)	0.0170 (13)	0.0142 (13)	0.0026 (10)	−0.0035 (10)	−0.0030 (11)
C15	0.0277 (15)	0.0130 (13)	0.0275 (17)	0.0024 (11)	−0.0097 (12)	0.0010 (12)
C16	0.0294 (15)	0.0179 (15)	0.0241 (16)	−0.0075 (11)	−0.0090 (12)	0.0058 (12)
C17	0.0225 (14)	0.0299 (16)	0.0145 (14)	−0.0085 (11)	0.0008 (10)	0.0059 (12)

### Geometric parameters (Å, °)

**Table:**

Br1—C12	1.873 (2)	C5—C6	1.387 (3)
Cl1—C14	1.713 (3)	C5—H5	0.9300
O1—C4	1.375 (3)	C6—H6	0.9300
O1—C7	1.430 (3)	C7—C8	1.510 (3)
O2—C9	1.222 (3)	C7—H7A	0.9700
N1—C9	1.350 (3)	C7—H7B	0.9700
N1—C1	1.419 (3)	C8—H8A	0.9600
N1—H1	0.877 (9)	C8—H8B	0.9600
N2—C12	1.322 (3)	C8—H8C	0.9600
N2—N3	1.367 (2)	C9—C10	1.488 (3)
N3—C10	1.376 (3)	C10—C11	1.367 (3)
N3—C13	1.429 (3)	C11—C12	1.391 (3)
N4—C17	1.330 (3)	C11—H11	0.9300
N4—C13	1.333 (3)	C13—C14	1.384 (3)
C1—C6	1.379 (3)	C14—C15	1.386 (4)
C1—C2	1.389 (3)	C15—C16	1.373 (4)
C2—C3	1.378 (3)	C15—H15	0.9300
C2—H2	0.9300	C16—C17	1.381 (4)
C3—C4	1.379 (3)	C16—H16	0.9300
C3—H3	0.9300	C17—H17	0.9300
C4—C5	1.384 (3)		
			
C4—O1—C7	116.83 (18)	C7—C8—H8A	109.5
C9—N1—C1	126.63 (19)	C7—C8—H8B	109.5
C9—N1—H1	117.9 (14)	H8A—C8—H8B	109.5
C1—N1—H1	115.4 (14)	C7—C8—H8C	109.5
C12—N2—N3	103.58 (17)	H8A—C8—H8C	109.5
N2—N3—C10	111.61 (18)	H8B—C8—H8C	109.5
N2—N3—C13	116.61 (17)	O2—C9—N1	125.03 (19)
C10—N3—C13	130.86 (17)	O2—C9—C10	120.47 (19)
C17—N4—C13	117.5 (2)	N1—C9—C10	114.5 (2)
C6—C1—C2	118.5 (2)	C11—C10—N3	106.54 (18)
C6—C1—N1	118.0 (2)	C11—C10—C9	133.6 (2)
C2—C1—N1	123.5 (2)	N3—C10—C9	119.8 (2)
C3—C2—C1	119.7 (2)	C10—C11—C12	104.7 (2)
C3—C2—H2	120.2	C10—C11—H11	127.6
C1—C2—H2	120.2	C12—C11—H11	127.6
C2—C3—C4	121.7 (2)	N2—C12—C11	113.5 (2)
C2—C3—H3	119.2	N2—C12—Br1	120.16 (16)
C4—C3—H3	119.2	C11—C12—Br1	126.31 (18)
O1—C4—C3	115.6 (2)	N4—C13—C14	123.2 (2)
O1—C4—C5	125.3 (2)	N4—C13—N3	114.5 (2)
C3—C4—C5	119.1 (2)	C14—C13—N3	122.1 (2)
C4—C5—C6	119.0 (2)	C13—C14—C15	118.3 (2)
C4—C5—H5	120.5	C13—C14—Cl1	120.6 (2)
C6—C5—H5	120.5	C15—C14—Cl1	121.1 (2)
C1—C6—C5	122.0 (2)	C16—C15—C14	119.0 (2)
C1—C6—H6	119.0	C16—C15—H15	120.5
C5—C6—H6	119.0	C14—C15—H15	120.5
O1—C7—C8	107.1 (2)	C15—C16—C17	118.5 (2)
O1—C7—H7A	110.3	C15—C16—H16	120.7
C8—C7—H7A	110.3	C17—C16—H16	120.7
O1—C7—H7B	110.3	N4—C17—C16	123.5 (3)
C8—C7—H7B	110.3	N4—C17—H17	118.2
H7A—C7—H7B	108.6	C16—C17—H17	118.2
			
C12—N2—N3—C10	1.4 (3)	N1—C9—C10—C11	5.7 (4)
C12—N2—N3—C13	171.6 (2)	O2—C9—C10—N3	6.4 (4)
C9—N1—C1—C6	−167.1 (2)	N1—C9—C10—N3	−172.7 (2)
C9—N1—C1—C2	13.5 (4)	N3—C10—C11—C12	1.4 (3)
C6—C1—C2—C3	−1.0 (4)	C9—C10—C11—C12	−177.1 (3)
N1—C1—C2—C3	178.3 (3)	N3—N2—C12—C11	−0.4 (3)
C1—C2—C3—C4	0.2 (5)	N3—N2—C12—Br1	−179.10 (15)
C7—O1—C4—C3	−174.8 (2)	C10—C11—C12—N2	−0.6 (3)
C7—O1—C4—C5	5.3 (4)	C10—C11—C12—Br1	177.93 (18)
C2—C3—C4—O1	−179.5 (3)	C17—N4—C13—C14	1.5 (3)
C2—C3—C4—C5	0.4 (5)	C17—N4—C13—N3	177.11 (17)
O1—C4—C5—C6	179.7 (2)	N2—N3—C13—N4	−69.3 (2)
C3—C4—C5—C6	−0.1 (4)	C10—N3—C13—N4	98.6 (3)
C2—C1—C6—C5	1.4 (4)	N2—N3—C13—C14	106.3 (2)
N1—C1—C6—C5	−178.0 (2)	C10—N3—C13—C14	−85.8 (3)
C4—C5—C6—C1	−0.8 (4)	N4—C13—C14—C15	−1.8 (3)
C4—O1—C7—C8	−179.8 (2)	N3—C13—C14—C15	−177.04 (19)
C1—N1—C9—O2	0.4 (4)	N4—C13—C14—Cl1	176.20 (16)
C1—N1—C9—C10	179.5 (2)	N3—C13—C14—Cl1	0.9 (3)
N2—N3—C10—C11	−1.8 (3)	C13—C14—C15—C16	0.7 (3)
C13—N3—C10—C11	−170.2 (2)	Cl1—C14—C15—C16	−177.25 (17)
N2—N3—C10—C9	176.95 (19)	C14—C15—C16—C17	0.5 (3)
C13—N3—C10—C9	8.6 (4)	C13—N4—C17—C16	−0.2 (3)
O2—C9—C10—C11	−175.3 (3)	C15—C16—C17—N4	−0.7 (3)

### Hydrogen-bond geometry (Å, °)

**Table:**

*D*—H···*A*	*D*—H	H···*A*	*D*···*A*	*D*—H···*A*
N1—H1···N4^i^	0.88 (1)	2.21 (1)	3.059 (3)	165 (2)
C2—H2···O2	0.93	2.26	2.850 (3)	121
C6—H6···N4^i^	0.93	2.60	3.358 (3)	140
C8—H8A···O2^ii^	0.96	2.45	3.397 (3)	168
C11—H11···N4^i^	0.93	2.55	3.390 (3)	151
C16—H16···O2^iii^	0.93	2.33	3.244 (3)	167

Symmetry codes: (i) *x*, −*y*+1/2, *z*+1/2; (ii) −*x*+1, −*y*+1, −*z*+2; (iii) *x*, −*y*+3/2, *z*−1/2.
